# A transgenic mice model of retinopathy of cblG-type inherited disorder of one-carbon metabolism highlights epigenome-wide alterations related to cone photoreceptor cells development and retinal metabolism

**DOI:** 10.1186/s13148-023-01567-w

**Published:** 2023-10-05

**Authors:** Karim Matmat, Jean-Baptiste Conart, Paul-Henri Graindorge, Sandra El Kouche, Ziad Hassan, Youssef Siblini, Rémy Umoret, Ramia Safar, Okan Baspinar, Aurélie Robert, Jean-Marc Alberto, Abderrahim Oussalah, David Coelho, Jean-Louis Guéant, Rosa-Maria Guéant-Rodriguez

**Affiliations:** 1 Inserm UMRS 1256 NGERE – Nutrition, Genetics, and Environmental Risk Exposure, University of Lorraine, 54500 Vandoeuvre-lès-Nancy, France; 2 National Center of Inborn Errors of Metabolism, University Regional Hospital Center of Nancy, 54000 Nancy, France; 3 Department of Ophthalmology, University Regional Hospital Center of Nancy, 54000 Nancy, France; 4 Faculté de Médecine, Bâtiment C 2Ème Étage, 9 Avenue de La Forêt de Haye, 54505 Vandœuvre-lès-Nancy, France

**Keywords:** Methionine synthase, DNA methylation, Epigenetics, Retina, Retinoid metabolism, Vitamin B12

## Abstract

**Background:**

*MTR* gene encodes the cytoplasmic enzyme methionine synthase, which plays a pivotal role in the methionine cycle of one-carbon metabolism. This cycle holds a significant importance in generating S-adenosylmethionine (SAM) and S-adenosylhomocysteine (SAH), the respective universal methyl donor and end-product of epigenetic transmethylation reactions. *cblG* type of inherited disorders of vitamin B12 metabolism due to mutations in *MTR* gene exhibits a wide spectrum of symptoms, including a retinopathy unresponsive to conventional therapies.

**Methods:**

To unveil the underlying epigenetic pathological mechanisms, we conducted a comprehensive study of epigenomic-wide alterations of DNA methylation by NGS of bisulfited retinal DNA in an original murine model with conditional *Mtr* deletion in retinal tissue. Our focus was on postnatal day 21, a critical developmental juncture for ocular structure refinement and functional maturation.

**Results:**

We observed delayed eye opening and impaired visual acuity and alterations in the one-carbon metabolomic profile, with a notable dramatic decline in SAM/SAH ratio predicted to impair DNA methylation. This metabolic disruption led to epigenome-wide changes in genes involved in eye development, synaptic plasticity, and retinoid metabolism, including promoter hypermethylation of *Rarα*, a regulator of *Lrat* expression. Consistently, we observed a decline in cone photoreceptor cells and reduced expression of *Lrat*, *Rpe65,* and *Rdh5*, three pivotal genes of eye retinoid metabolism.

**Conclusion:**

We introduced an original in vivo model for studying *cblG* retinopathy, which highlighted the pivotal role of altered DNA methylation in eye development, cone differentiation, and retinoid metabolism. This model can be used for preclinical studies of novel therapeutic targets.

**Supplementary Information:**

The online version contains supplementary material available at 10.1186/s13148-023-01567-w.

## Background

*MTR* gene encodes the cytoplasmic enzyme methionine synthase (MS), which plays a pivotal role in the methionine cycle of one-carbon metabolism. MS requires the methyl-cobalamin coenzyme form of vitamin B12 as a cofactor to catalyze the conversion of homocysteine (HCY) to methionine [1]. The resulting methionine is then used as a precursor for the synthesis of the universal methyl donor, S-Adenosylmethionine (SAM), which enables methylation of various biomolecules including proteins, nucleic acids, and other metabolites [2].

Genetic mutations affecting the *MTR* gene are responsible for a rare inherited error of cobalamin metabolism (IECM) called homocystinuria megaloblastic anemia *cblG* group (OMIM #250,940). This disease is characterized at the metabolic level by an increased level of HCY and a low methionine level [2, 3]. Symptoms in patients are multiple and depend on the mutation pathogenicity [3]. The most frequent outcomes include neurological manifestations, severe megaloblastic anemia, and ophthalmological disorders [3, 4]. Neurological and ocular abnormalities are common, with decreased visual acuity, nystagmus, optic atrophy, strabismus, and retinal anomaly which are linked to rod and cone cell dysfunction [4, 5]. Current treatments are essentially aimed at correcting metabolic abnormalities and are based on high-dose injections of hydroxocobalamin to stimulate the residual activity of MS [6, 7]. This treatment demonstrates promising effectiveness in managing metabolic and hematological symptoms. However, its impact on neurological and ocular symptoms as well as their progression is limited and can potentially result in visual impairment that may progressively lead to a significant decline in visual acuity [4].

Although no study has specifically investigated the consequences of MS deficiency on the retina, several studies have highlighted the detrimental effects of vitamin B12 deficiency, which is a cofactor for MS, on vision and the retina. Indeed, B12 deficiency leads to a decreased retinal nerve fiber layer thickness, vascular changes, inflammation, and decreased visual acuity [8–11].

Recent published transcriptomic data on fibroblast of *cblG* patients [12] highlighted a dysregulation in the expression of several genes involved in the retinal ganglion cell axon guidance process. Other studies in patients’ fibroblasts, neuron-like cells, and transgenic mice with impaired vitamin B12 cellular availability have shown that decreased MS activity produces dramatic effects on gene expression through epigenomic mechanisms that include altered DNA methylation [2].

A better understanding of the pathomechanisms of MS deficiency is necessary for the development of therapeutic approaches for ocular symptoms in *cblG* pathology. However, since *Mtr* knockout is lethal and experimental studies conducted on rats with vitamin B12 deficiency cannot rule out other effects related to nutritional deficiencies, careful consideration of alternative experimental models was needed. To specifically investigate the effects of *Mtr* silencing on the eye and the brain, we generated a transgenic mouse model with the targeted disruption of *Mtr* expression in those regions [13, 14]. We examined the behavioral, epigenomic, and cellular effects of this selective MS deficit in the retina using this model. We conducted metabolomic and methylome analyses on retinal tissue to gain insight into the epigenetic, metabolic, cellular, and molecular mechanisms underlying the ocular manifestations of *cblG*-type inherited disorders of vitamin B12 metabolism.

## Results

### The selective silencing of *Mtr* expression by Cre-Lox produces dramatic metabolic changes and a drop in SAM/SAH ratio in the retina

**Fig. 1:**
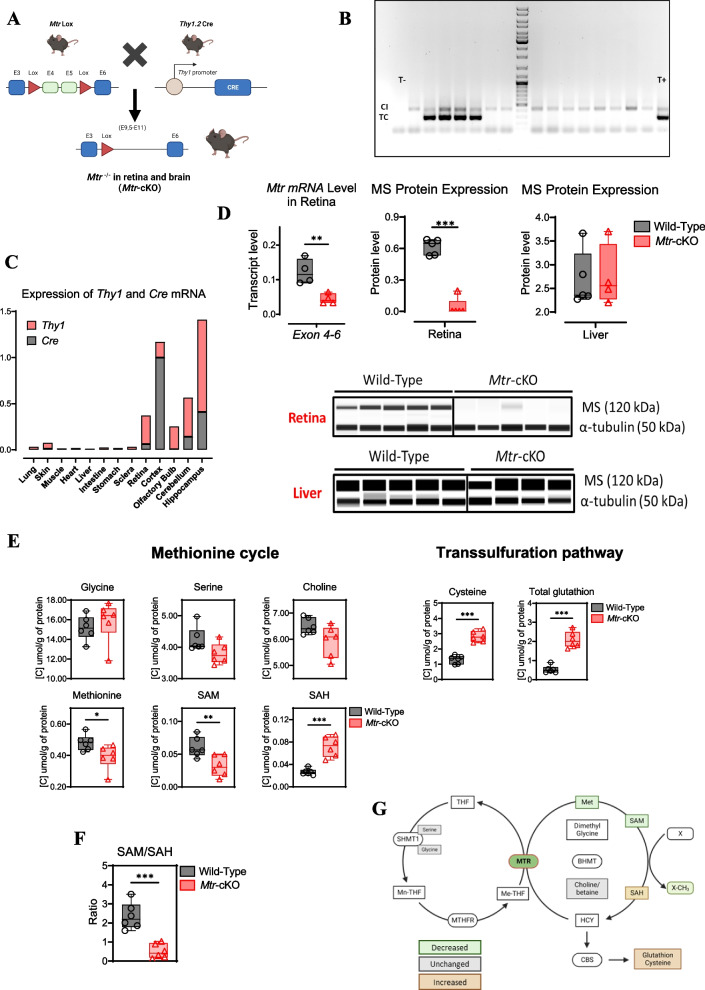
Invalidation of *Mtr* reduces methionine synthase expression in the retina and disrupts one-carbon metabolism and remethylation pathway resulting in decreased SAM/SAH ratio and enhanced transsulfuration pathway activity. **A** Genetic model of *Mtr*-cKO. Mice expressing the Cre recombinase under the control of the *Thy1* promoter are bred with mice carrying floxed exons 4 and 5 of the *Mtr* gene (indicated by red triangles). Offspring from this cross exhibit conditional deletion of the *Mtr* gene (Created with BioRender.com). **B** Representative PCR analysis of the Cre gene in tail extracts obtained on postnatal day 15 (CI: internal control; TC: Cre gene; T + : positive control; T-: negative control). **C** Quantification of mRNA levels of *Thy1* and *Cre* using RT-qPCR in several tissues. **D** Quantification of mRNA and protein levels of the *Mtr* gene and methionine synthase (MS) protein in retinal and liver tissue using RT-qPCR and WES Simple Protein. α-tubulin serves as an internal control for protein expression, and densitometric analysis of the WES assay provides quantification of protein expression. **E** Analysis of one-carbon metabolites in retinal tissue using LC/MSMS. **F** SAM/SAH ratio in the retina. **G** Schematic representation of altered one-carbon metabolism. Increased protein/metabolite expression/concentration is indicated in orange, decreased in green, and unchanged in gray (Created with BioRender.com). Data are presented as means ± SEM. Statistical analysis was performed using the Student t test (*P < 0.05, **P < 0.01, ***P < 0.001). *N* = 4 to 6 per group

Mice from the *Mtr*-cKO group was identified by genotyping (Fig. 1A and B**)**. The specificity of *Mtr* deletion was confirmed by the analyzed of *Thy1* and *Cre* mRNA level in several tissues. We observed a positive expression of *Thy1 and Cre* expression in retina and neuronal tissue, while several organs such as liver show no expression (Fig. 1C). Mice from the *Mtr*-cKO group exhibited a significant reduction in both transcript and protein levels of MS in the retinas compared to the wild-type group. However, when analyzing MS expression in the liver, no significant differences were observed (Fig. 1D**).**

We further investigated the metabolic consequences of the *Mtr* gene deletion in the retinal tissue. We found that *Mtr* deletion produced a significantly decreased level of methionine and SAM, while S-adenosylhomocysteine (SAH) levels exhibit an increased concentration in the *Mtr*-cKO group in comparison with the wild-type animals (Fig. 1E). Hcy level could not be measured due to his low concentration in the retina. Interestingly, we found an increase in the transsulfuration pathway which was evidenced by the higher levels of both cysteine and total glutathione in the *Mtr*-cKO group (Fig. 1E). Because of the impaired synthesis of methionine, the SAM/SAH ratio (indicative index of transmethylation reactions) is significantly decreased in the *Mtr*-cKO group compared to the control mice (Fig. 1F). These results are consistent with the blockage of the remethylation pathway through the MS (Fig. 1G).

### Retinal MS deficiency causes delayed eye opening and impaired visual acuity despite normal eye fundus

**Fig. 2:**
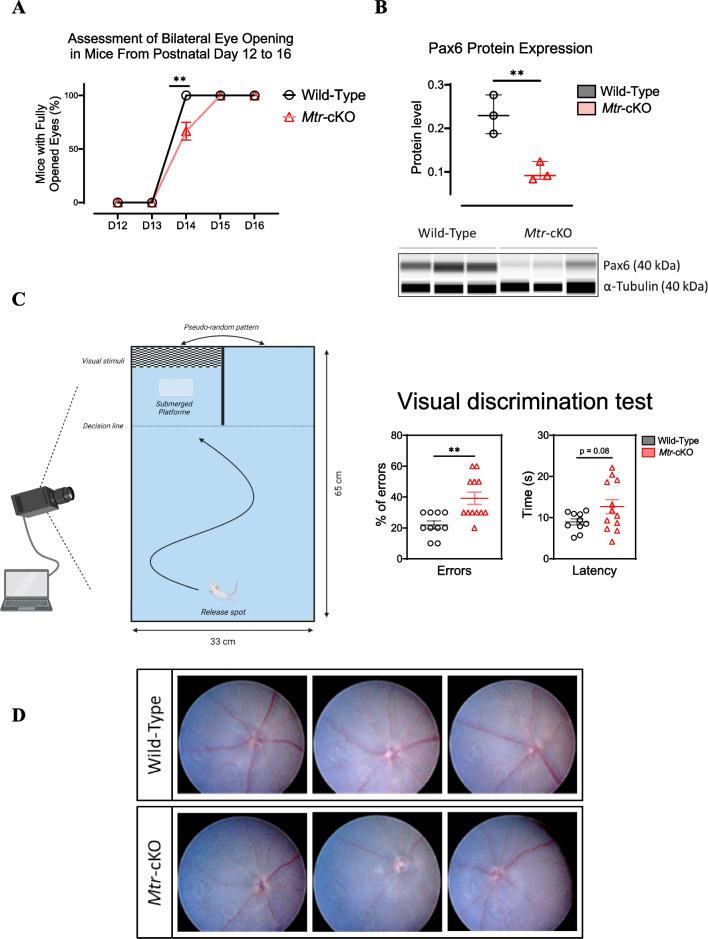
Ocular phenotyping of *Mtr*-cKO mice and fundus examination. **A** Assessment of bilateral eye opening from postnatal day 12 to 16. **B** Quantification levels of Pax6 protein in retinal tissue using WES Simple Protein. α-tubulin serves as an internal control for protein expression, and densitometric analysis of the WES assay provides quantification of protein expression. **C** Schematic representation of the visual acuity test conducted on postnatal day 21 and visual acuity test results including the percentage of failures during the test and latency to find the platform. *N* = 12/group. Data are presented as means ± SEM. Statistical analysis was performed using Student t test (*P < 0.05, **P < 0.01, ***P < 0.001). (Created with BioRender.com). **D** Fundus examination of the dilated eye using the AIDA Compact II system. Data represent means ± SEM. Statistical analysis was performed using the Student t test (*P < 0.05, **P < 0.01, ***P < 0.001). *N* = 3 to 12 mice per group

We investigated the implications of retinal MS deficiency on ocular development and visual acuity. By evaluating the age of both eyes opening in *Mtr*-cKO mice compared to the control group at very early postnatal age, we identified a significant delay in the opening of both eyes. This delay, notably observed on postnatal day 14, is indicative of potential disruptions in the developmental progression of the eyes within the deficient mice (Fig. 2A). The delay in eye development was also shown by a decreased in the expression of Pax6 protein in the retina, a major transcription factor involved in the eye development at embryonic stage and development of retina and optic nerve at postnatal stage (Fig. 2B). However, the visual acuity test (Fig. 2C) illustrates a decrease in visual acuity among *Mtr*-cKO mice when compared to the wild-type group. On day 21 (D21), *Mtr*-cKO mice recorded a significantly higher error percentage during the test than control mice, indicative of mildly impaired visual acuity. However, no significant difference emerged in the time taken to reach the platform from the starting point, with only a trend toward increased latency noted in the *Mtr*-cKO group (*p* = 0.08) (Fig. 2C). Furthermore, to validate our findings of visual acuity alteration on KO group, we did several validations tests, where *Mtr*-cKO mice have demonstrated no abnormalities associated with striatum-dependent learning, as compared to the wild-type group. This was consistent across both learning tests and the prob-test; the latter revealed an equal (50–50%) choice for right and left side in the absence of visual stimulus (data not shown). A fundus examination revealed no significant retinal anomalies. However, several *Mtr*-cKO mice exhibited reduced vascularization, although this finding varied among individual mice (Fig. 2D). This interindividual variability aligns with the symptoms observed in patients with *cblG*-type cobalamin deficiency.

### *Mtr*-conditional Knock-Out mice exhibit alterations in genome-wide DNA methylation in the retina related to eye development, retinoid metabolism, and synaptic plasticity in the retina

To have a better overview of dysregulated mechanisms induced by the drop in the SAM/SAH ratio, we carried out methylome analyses of the retinal tissue to decipher the epigenetic consequences of the *Mtr* deletion.

**Fig. 3:**
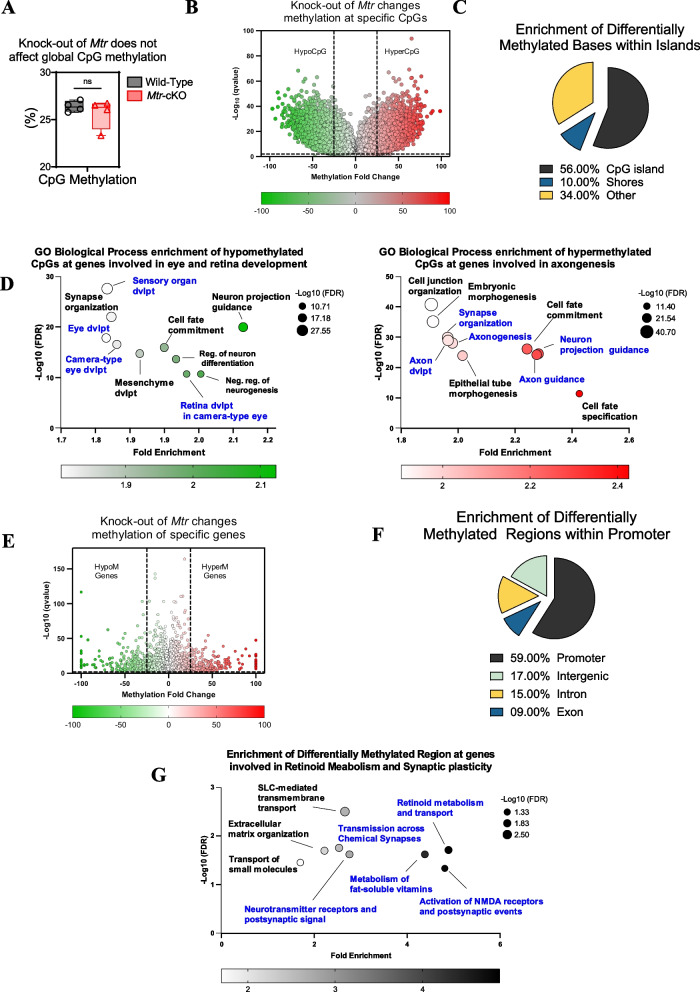
Genome-wide DNA methylation analysis in the retina of 21-day-old mice. **A** *Mtr* deficiency does not change global CpG methylation. **B** Volcano plot highlighting *Mtr* deficiency-induced differentially methylated CpGs (methylation difference > 25 and *q* < 0.01). Among the differentially methylated CpGs the decreased methylated CpGs are represented in green (HypoCpG) and increased methylated in red (HyperCpG). **C** Genomic enrichment of differentially methylated CpGs. HypoCpG and hyperCpG are mainly located at CpG islands. **D** Top 10 most significantly enriched Gene Ontology (GO) biological process terms for hypo- and hypermethylated CpG sites in the retina of *Mtr*-cKO mice, respectively represented in green and red (FDR < 0.05). **E** Volcano plot highlighting *Mtr* deficiency-induced differentially methylated regions (methylation difference > 15 and *q* < 0.01). Among the differentially methylated regions the decreased methylated are represented in green (HypoM Genes) and increased methylated in red (HyperM Genes). **D** Genomic enrichment of differentially methylated regions. HypoM and HyperM genes are mainly located at the promoter. **G** Top 10 most significantly enriched Reactome Pathways Database terms for DMRs in the retina of *Mtr*-cKO mice (FDR < 0.05). **E** Venn diagram highlighting differentially methylated genes involved in eye development and their corresponding methylation fold change. **F** Venn diagram illustrating differentially methylated genes associated with retinoid metabolism and their corresponding methylation fold change

While the *Mtr* deficiency does not change global CpG methylation (Fig. 3A), volcano plots highlight specific differentially methylated CpGs [methylation difference > 25 and *q* < 0.01]. Among the differentially methylated CpGs, 50.86% exhibit decreased methylation (hypoCpG—Green) and 49.14% increased methylation (hyperCpG—Red) in *Mtr*-cKO mice and this differentially methylated CpGs was predominantly clusters within CpG islands (Fig. 3B and C). Top 10 “Gene Ontology—Biological Process” terms show that hypomethylated CpG is involved in process related to “Sensory organ development,” “Eye development,” “Camera-type eye development,” or “Retina development in camera-type eye” (Fig. 3D), while GO analysis of hypermethylated CpG highlights term related to “Synapse organization,” “Axogenesis,” “Neuron projection guidance,” or “Axon development” (Fig. 3D).

The Analyses of Differentially Methylated Regions (DMRs) highlight 1084 unique significant DMRs. Indeed, volcano plots show specific DMRs [methylation difference > 15 and *q* < 0.01]. Among the DMRs, 50.28% exhibit decreased methylation (HypoM Genes—Green) and 49.72% increased methylation (HyperM Genes—Red) in *Mtr*-cKO mice and this DMRs was predominantly clusters within promoter regions (Fig. 3E and F). The Top 10 pathways enrichment using “Reactome Pathway Database” terms shows that differentially methylated genes are involved in processes related to “SLC-mediated transmembrane transport,” “Transmission across Chemical Synapses,” “Activation of NMDA receptors,” and “Retinoid metabolism and transport” (Fig. 3G**)**.

Overall, the analysis of differentially methylated CpG sites and differentially methylated regions (DMRs) revealed significant DNA methylation changes associated with eye and retina development, synaptic plasticity, and vitamin and retinoid metabolism which is a crucial process for visual phototransduction in the retina.

To identify the genes involved in those processes, we utilized the MGI database (https://www.informatics.jax.org/) that catalogs mouse genes associated with “Eye development #GO:0001654” and “Retinoid metabolism process #GO:0001523.”

**Fig. 4:**
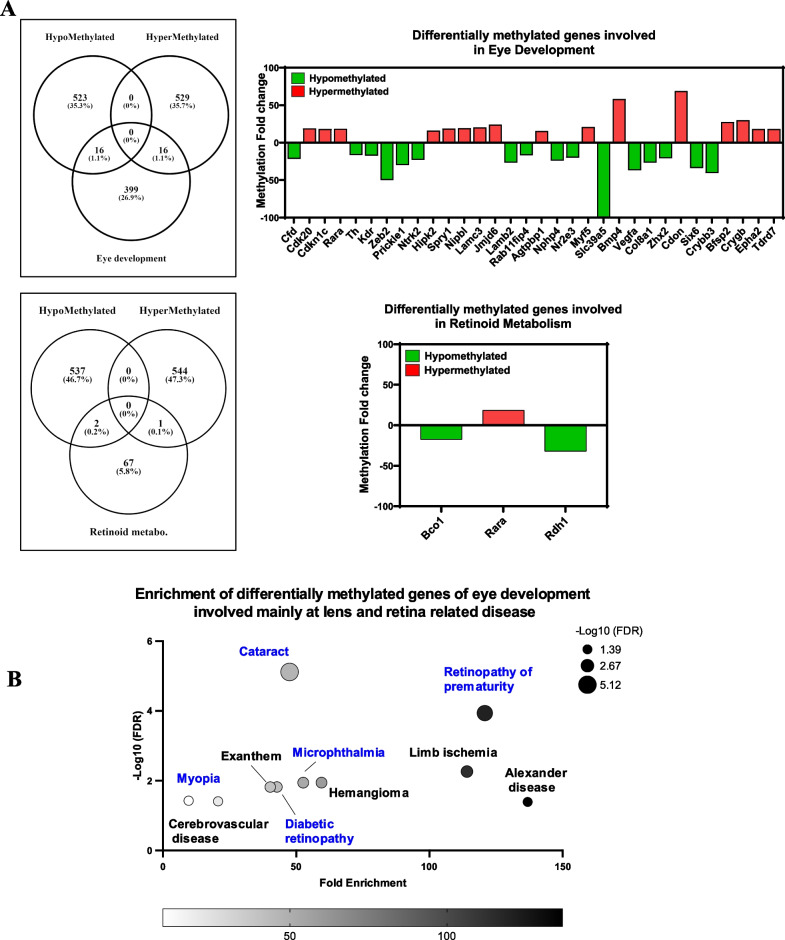
Specific genes methylation changes related to eye development and retinoid metabolism are mainly. **A** The Venn diagram shows 32 differentially methylated genes related to eye development with 16 hyper and 16 hypomethylated genes. At the same manner, the Venn diagram 3 differentially methylated genes related to retinoid metabolism with 1 hyper- and 2 hypomethylated genes. **B** Top 10 most significantly enriched JENSEN Disease terms for differentially methylated genes involved in eye development and retinoid metabolism (FDR < 0.05)

Using a Venn diagram (https://bioinfogp.cnb.csic.es/), we summarized the overlap of differentially methylated genes involved in eye development and retinoid metabolism processes, along with their corresponding methylation fold changes in the retina of *Mtr*-cKO mice compared to the control group (Fig. 4A). This comprehensive visualization provides insights into the specific genes that are affected in these critical biological pathways and their corresponding methylation changes in the context of *Mtr*-cKO mice. Among those genes we found an hypermethylation of Retinoic Acid Receptor alpha (*Rarα)* a specific regulator of retinoid-related genes in the retina, an hypomethylation of *Vegfa*, a crucial regulator of angiogenesis, that can be related to the vascular changes observed in eye fundus, an hypermethylation of *Epha2* which is involved in axon guidance and an hypomethylation of *Nr2e3* which is a crucial well-known regulator of cone differentiation. Enrichment analysis using “The JENSEN Disease database” indicates that these differentially methylated genes are mainly involved in specific ocular phenotypes including “Cataracts, Retinopathy of Prematurity, Myopia, Microphthalmia and Diabetic Retinopathy” (Fig. 4B).

### The MS deficiency led to an alteration of major genes of retinoid metabolism

**Fig. 5:**
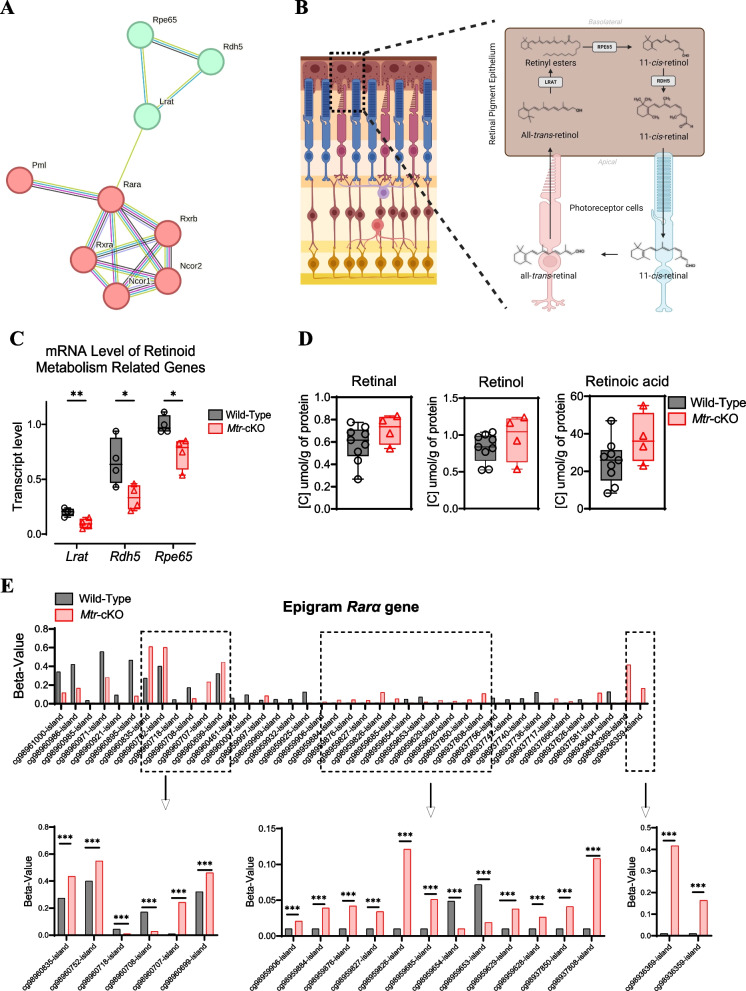
Analysis of retinoid metabolism and upstream regulator. **A** Graph of the STRING software interaction analysis. Analysis of the interaction and upstream regulation of the 3 main retinoid-related genes (*Lrat*, *Rpe65*, and *Rdh5*) using STRING software highlight the involvement of *Rarα* in the regulation of *Lrat*. **B** Schematic representation of the retinoid metabolism in the retina, emphasizing the three main enzymes involved in the cycle. **C** Quantification of mRNA levels for *Lrat*, *Rpe65*, and *Rdh5* genes in retinal tissue using RT-qPCR. **D** Analysis of retinoid metabolites in retinal tissue using LC/MSMS approach. **E** Epigenetic analysis displaying the beta values of significantly differentially methylated CpGs positions in the promoter of *Rarα* gene in *Mtr*-cKO mice compared to wild type. Data represent means ± SEM. Statistical analysis was performed using the Student t test (*P < 0.05, **P < 0.01, ***P < 0.001). *N* = 3 to 5 mice per group

Regarding the previous results, we further investigate the retinoid metabolism, which is a crucial process for retinal function and visual phototransduction. We analyzed the mRNA level of major enzymes of retinoid metabolism in the retina that are regulated by Rarα (Fig. 5A and B). The expression results of *Lrat*, *Rpe65,* and *Rdh5* which are 3 of the main enzymes involved in the conversion of all-trans-retinol into 11-cis retinal highlighted a significantly decreased transcription in the retina of *Mtr*-cKO mice (Fig. 5C). However, *Lrat*, *Rpe65,* and *Rdh5* genes promoters did not show any methylation changes despite the decreased mRNA expression (data not shown), suggesting the possible upstream mechanisms regulation through Rarα. Moreover, metabolomic analysis of retinoid metabolites in the retina did not show any difference in the concentration of total retinol, retinal, and retinoic acid between the two groups despite the decreased transcription of the genes **(**Fig. 5D**)**, suggesting a possible concentration changes of specific forms of retinol and/or retinal (specifically 11-cis retinal/retinol and/or all-trans-retinal). Notably, as discussed before, the methylome analysis highlights a significant hypermethylation of the promoter of *Rara* gene. The nuclear receptor Rarα encoded by this gene is a well-known positive regulator of *Lrat* expression (Fig. 5E and Additional file 1: Fig. S2).

### The invalidation of MS in the retina leads to an isolated decreased cone population

**Fig. 6:**
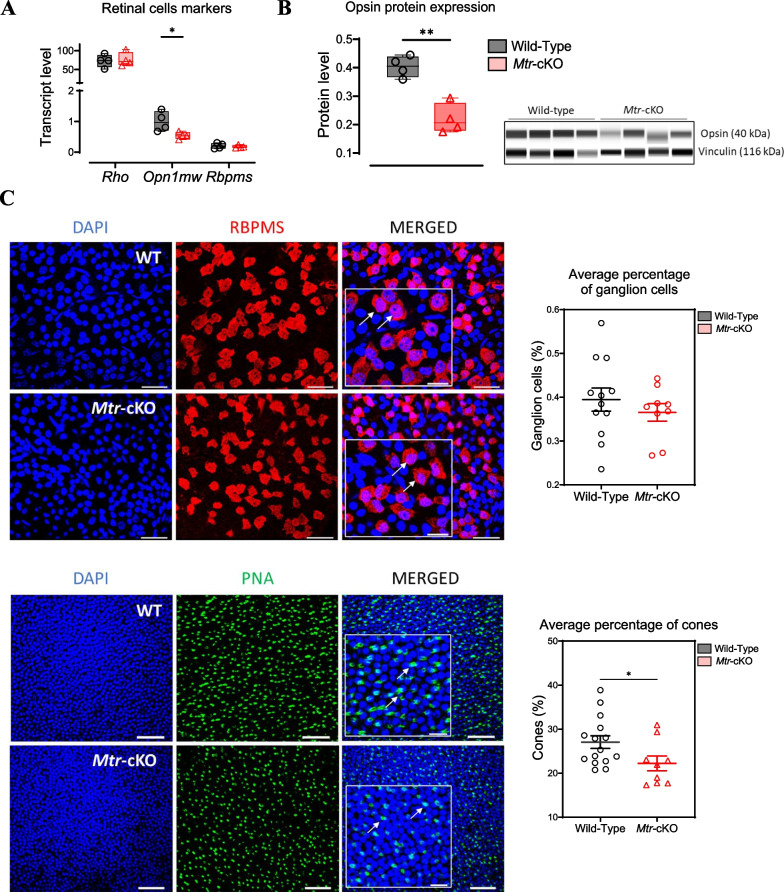
Analysis of retinal cell type in *Mtr*-cKO mice compared to wild type. **A** Quantification of mRNA levels for *Rho*, *Opn1mw*, and *Rbpms* genes in retinal tissue using RT-qPCR. **B** Analysis of protein levels for Opsin protein in retinal tissue using WES Simple Protein. Vinculin was employed as an internal control for protein expression. Densitometric analysis of the WES assay provides quantification of protein expression. **C** Immunostaining of retinal ganglion cells and cones using specific labeling for RBPMS and PNA, respectively, on flat-mounted retinas of wild type and *Mtr*-cKO mice. Examples of labeling are indicated by arrows. The scale bars in the main pictures represent 10 µm, while the scale bars in the insets denote 5 µm. The average percentages of retinal ganglion cells and cones are presented in the histograms. Data represent means ± SEM. Statistical analysis was performed using the Student t test (*P < 0.05, **P < 0.01, ***P < 0.001). *N* = 3 to 5 mice per group

Analysis results of specific markers of rods, cones, and retinal ganglion cells using RT-qPCR show a decreased mRNA level of *Opn1mw* (encoding for Opsin, a specific marker of cone population) in the retina of *Mtr*-cKO mice, while the mRNA level of *Rho* and *Rbpms*, which are markers for rods and retinal ganglion cells, respectively, showed no differences (Fig. 6A).

The decreased expression of Opsin was confirmed at the protein level. Results indicate a significantly reduced expression in the retina of *Mtr*-cKO mice (Fig. 6B). RBPMS as well as Rhodopsin protein levels do not change significantly between the 2 groups (Additional file 1: Fig. S1). Those results are consistent with immunofluorescence of the flat-mounted retina. Indeed, the results show fewer number of PNA^+^ cones while retinal ganglion cells RBPMS^+^ show no difference (Fig. 6C).

## Discussion

In *cblG* patients, the ocular phenotype includes decreased visual acuity, optic nerve atrophy, and photoreceptor-related retinal dysfunction [4, 5]. The current standard of care is OHCbl injection, which usually results in a decrease in HCY and an increase in methionine concentration, implying a restoration and stabilization of metabolic parameters. However, a longitudinal follow-up study of *cblG* and also of other IECM patients indicate that treatments appear to have little to no effect on the ocular phenotypes which persist and progress despite treatment [4, 15, 16]. While the underlying pathomechanisms of ocular symptoms remain elusive, there is a lack of studies that specifically address the origin of these symptoms in *cblG* disorders from a retinal-focused perspective, and this can be attributed largely to the absence of suitable experimental models. Indeed, studying the ocular consequences of reduced or absent MS activity presents a significant challenge, given the unavailability of patient retinal tissue and the fact that systemic *Mtr* knockout proves to be lethal in mouse models. To address this issue, we have developed the first suitable transgenic mouse model exhibiting an *Mtr* gene deletion in the retina and brain achieved by utilizing the controlled expression of Cre recombinase under the *Thy1.2* promoter. This model allowed us to specifically study the ocular consequences of the impaired remethylation pathway in the retina. In the mouse retina, the *Mtr*-cKO mice exhibited a strongly decreased level of *Mtr* mRNA and MS protein expression compared to the control group. We found major metabolic changes related to one-carbon metabolism pathways to a level that is comparable to those found in *cblG* patients [6] but also in rats fed with a methyl donor deficiency diet (MDD) [17]. Indeed, methionine and SAM levels were significantly lower, SAH levels were significantly higher in the retina of *Mtr*-cKO mice, and the SAM/SAH ratio—a measure of transmethylation reactions—was significantly and dramatically decreased in the *Mtr*-cKO group compared to the control mice. However, the transsulfuration pathway appears to be overactivated, indicating that remethylation of HCY to methionine is impaired.

Despite the absence of major abnormality in the fundus examination which is concordant with data on *cblG* patients [5], *Mtr*-deficient mice exhibit a delay in the opening of both eyes, specifically at postnatal day 14 that has been corrected at day 15, giving that baby mice open their eyes at around the age of 13 days [18]. This delay may arise from impaired maturation of the visual system, as evidenced by the downregulation of Pax6 expression; indeed, this latter is a critical transcription factor involved in the embryonic and postnatal stages of visual system development and maturation [19, 20]. However, deficient mice also exhibit a slight decrease of visual acuity with some individual variability, similarly to the decreased visual acuity found in some IECM patients [5, 15]. In the methylome study, mice exhibiting the most significant decrease in visual acuity, compared to wild type, showed changes in methylation in the promoter regions of genes involved in eye development and retinoid metabolism which is crucial for visual phototransduction pathway [21]. These genes including specific genes involved in the formation of the lens, development of cone photoreceptors, and retinoid metabolism are mainly related to specifics ocular pathologies including cataracts, myopia, and retinopathy. While myopia and retinopathy are commonly found in the case of IECM, interestingly, cataract and lens abnormalities have not been reported [4]. Commonly, crystalline lens dislocation is associated with cystathionine β-synthase (CBS) deficiency, a condition characterized by severe hyperhomocysteinemia [22]. Although our study did not examine lens structure, subsequent investigations should probe potential lens abnormalities, including structural impairment, and their potential contribution to decreased visual acuity.

Although we did not observe differences in retinoid metabolite concentrations, the mRNA levels of three key visual cycle enzymes—*Lrat*, *Rpe65*, and *Rdh5*—were found to be reduced in the retinas of *Mtr*-cKO mice. Interestingly, the decreased gene expression does not correlate with the methylation of their promoters. Instead, we discovered a significant hypermethylation in the promoter region of *Rarα*, a known positive regulator of *Lrat* gene expression [23]. Indeed, the visual cycle-related gene *Lrat* is transcriptionally up-regulated in response to Am580 (RARα agonist) and to all-*trans*-retinal (AtRAL) treatments [23]. Interestingly, these treatments did not impact the expression of RPE65 and RDH5. This suggests that these two visual cycle genes are regulated differently than LRAT in a RARα independent manner. However, our data point to an epigenetic mechanism responsible for the decreased expression of the *Lrat* gene, a critical enzyme in retinoid metabolism, which might play a role in the ocular phenotype of *cblG* patients. Indeed, patients exhibiting mutations affecting *LRAT* and *RPE65* are responsible for dramatic progressive ocular diseases called Leber congenital amaurosis and Retinitis pigmentosa, and these pathologies are characterized by several retinal dystrophies [24, 25]. Studies on mice lacking the *Lrat* gene have an early loss of rod and cone photoreceptors, while mice lacking the *Rpe65* gene exhibit loss of photoreceptors and early cone degeneration [26]. Similarly, retinal cell analysis in *Mtr*-cKO mice demonstrated a marked reduction in the expression of cone photoreceptor markers, and fewer number of cone cells throughout the retina at early postnatal stage (day 21) compared to wild-type mice. These results could be a direct consequence of impaired retinoid metabolism which could lead to early and progressive cone degeneration. A future study will investigate the effects of reduced *Lrat* mRNA levels, focusing on the specific retinoid metabolite, 11-cis retinal, and analyzing the apoptosis state in the retina and lipofuscin level, a well-known biomarker for retinal cells degeneration. Indeed, mice deficient in *Lrat*, displaying lower levels of 11-cis retinal, experience cone degeneration. However, supplementation with 11-cis retinal can restore cone response in these mice, indicating a vital role of *Lrat* and 11-cis retinal in maintaining cone homeostasis [26]. Conversely, methylome study underscored hypomethylation in the *Nr2e3* gene promoter. As the retina-specific nuclear receptor *Nr2e3* is known for repressing cone differentiation, this finding suggests potential developmental impairment that could contribute to the reduced cone cell population, indicative of a neurodevelopmental anomaly [27].

Interestingly, we noted no change in the population of retinal ganglion cells, even though optic nerve defects are commonly observed in *cblG* patients with ocular impairment [4]. However, methylome study highlighted hypermethylation of the *Epha2* gene. This finding complements a transcriptome study on *cblG* patient fibroblasts, which revealed dysregulated expression of *EPHA5* [12]. Importantly, both of these genes play a key role in guiding optic nerve axons [28]. These findings could point to a functional impairment in the myelination pathway rather than retinal ganglion cell degeneration. While this first study did not specifically target the optic nerve, this *Mtr*-cKO mouse model holds promise as a valuable tool for conducting in-depth investigations into optic nerve but also the myelination pathway impairments associated with MS deficiency. Such in vivo model could offer profound insights into the anomalies observed in patients with *cblG* disorders.

IECM is also often characterized by severe neurological impairment parallel to ocular symptoms. These neurological defects include brain atrophy, demyelinated area, and EEG abnormality [4, 15]. However, studies on rats showed that methyl donor deficiency that is characterized by a drop in SAM/SAH ratio is often linked to neuronal cell death, pro-apoptotic state, metabolic alteration, development and cell differentiation impairment, and synaptic alterations [29–31] which could affect visual cortex processing thus participating to the decrease visual acuity as reported in animals’ models with altered visual cortex [32–34]. This hypothesis will be investigated in a future study focusing on brain defects related to visual pathway through the deep examination of the epigenetic, metabolic, cellular, and molecular changes related to the visual cortex, providing a better knowledge of the multiple causes leading to ocular symptoms in *cblG* pathology.

Ocular abnormalities frequently manifest in *cblG* IECM cases. The retina, a pivotal structure for vision, serves as the core of the visual pathway. This first study, utilizing a novel in vivo model of substantial worth, has revealed that during the early stages of development, *Mtr* deletion results in a slight reduction in visual acuity along with changes in methylation within genes implicated in eye development, metabolism, and vision. In summary, *Mtr* silencing induces disruptions in one-carbon metabolism and remethylation pathway, genes associated with retinoid metabolism, and epigenetic modifications linked to DNA methylation. While further investigation is necessary to deepen our understanding of visual symptoms in *cblG* pathology, this initial study showcases the creation of a first distinctive, suitable, and innovative model. This model holds significant promise for exploring the detrimental impacts of MS deficiency, potentially paving the way for identifying novel avenues and therapeutic targets aimed at correct or ameliorating visual symptoms in *cblG* disease.

## Methods

### Animals and tissue collection

Experiments were performed at 19- to 21-day-old mice (C57BL/6) *Mtr*-knock-out (*Mtr*-cKO) in retina tissue, achieved by Cre/Lox system using *Thy-1.2* as a promoter of Cre recombinase. Animals have been treated following the National Institute of Health Guide for the Care and Use of Laboratory Animals, in an accredited establishment (Institut National de la Santé et de la Recherche Médicale, U1256) according to the UE guidelines 2010–63-UE and to French governmental decree 2013–118 and the authorization number Apafis #12,851. Mice were euthanized at D21 with an overdose of isoflurane. Tissues were rapidly harvested and kept frozen in liquid nitrogen and then stored at − 80 °C until biochemical/molecular and LC–MS/MS analyses or otherwise used directly for experiments that need fresh tissues.

### Behavioral testing

#### Assessment of bilateral eyelids opening and visual acuity test

We followed the pups on days 12, 13, 14, 15, and 16 of their lives, three times a day until the full eyes are open, to register any eyes development delay. The visual acuity performances were evaluated using a behavioral test adapted from Prusky et al. [35]. Behavioral observations of 12 wild-type and 12 *Mtr*-cKO mice (including 6 females and 6 males per group) were conducted using a video-tracking system (Viewpoint) allowing a high standardization between runs.

A rectangular swimming pool of 65 cm long and 33 cm wide, at the end of which are two corridors, was used and adapted for carrying out the visual acuity test. The pool was filled with water (25 °C) to a height of 3 cm allowing a walking swim. A platform hidden in the water and/or a visual stimulus, corresponding to a checkerboard whose squares are 15 mm in length, could be added.

The visual acuity test involves the vision, the dorso-median striatum, and the dorsolateral striatum [36]. This test involves stimulus–response association memory, that is, the memory that prompts the animal to go in a direction based on an understanding of an association between the visual stimulus and the platform. To carry out this test, each lane of the swimming pool was deposited or not a platform and a visual stimulus. This test requires a preliminary training stage, carried out at D20, in which the animal will try to assimilate that the platform is in the same place as the visual stimulus, regardless of the location. For the training stage, the visual stimulus and the platform are placed in the two lanes of the swimming pool, and then, the mouse is placed in front of the platforms. When the mouse reaches the platform, it is moved to the location of the reward (a heated cage). During this training step, each mouse undergoes 10 passages. After twenty-four hours allowing memory consolidation, the visual acuity test is performed (D21). During this test, the platform and the visual stimulus are placed in only one corridor, and their location is alternated between each pass following a pseudorandom pattern. Thus, the mouse is placed in front of the corridors (release spot) where it makes its choice between the left or right corridor based on the visual recognition of the stimulus. During this step, each mouse undergoes 19 passages, and 2 parameters are considered: the ability to find the platform directly (success) or not (failure) and the latency for the mice to get to the platform from the release spot.

### Eye fundus

The observation of the fundus is done externally on the animal anesthetized under isoflurane. It is a noninvasive method of exploration and imaging allowing the observation of the retina. The examination is performed under general anesthesia, and an endoscopic probe is used to view the retina after the pupil is dilated with Tropamide solution (Mydriaticum 2 mg/0.4 mL). The probe is positioned and guided by an experienced operator for a comprehensive examination of the retina. The endoscope is a mobile device, AIDA Compact II system (Karl Storz) adapted to the study of small rodents [37]. The endoscope is equipped with a light source, an optical system utilizing optical fibers, and various channels for operator control. The images (videos or photographs) are digitized and then analyzed by an ophthalmologist.

### Methylome analysis

For methylome studies, four retina samples were used per group. Genomic DNA was extracted using QIAamp DNA Kits (Qiagen) following the manufacturer’s instructions. Tissue lysis was performed overnight at 56 °C, and the DNA was eluate in 50 µl elution buffer. DNA concentration was measured using the Quant-iT™ PicoGreen™ dsDNA Assay Kit (Invitrogen), and DNA quality was analyzed using TapeStation instrument (4150 Agilent) and genomic DNA ScreenTape Assay kit (Agilent).

An aliquot of 100 ng of DNA was utilized for library preparation, bisulfite conversion, and amplification using the Diagenode Premium RRBS Kit (Liege, Belgium) as per the manufacturer’s guidelines. A total of 8 samples were mixed before the bisulfite conversion step. We used the methylated and unmethylated spike-in controls included in RRBS Diagenode kit to estimate the bisulfite conversion efficiency. The results indicated no over-conversion as the conversion rate of the methylated spike-in was below 2% and no under-conversion as the conversion rate of the unmethylated spike-in was above 98% as shown in Additional file 1: Table S1. Every library pool was quantified using Quant-iT™ PicoGreen™ dsDNA Assay Kit (Invitrogen), and the average size of DNA fragments was estimated with a 2100 Bioanalyzer instrument (Agilent) using High Sensitivity DNA Kit (Agilent). Every pool was then denatured in NaOH and diluted at 1.8 pm with 20% of PhiX Control v3 (Illumina). Sequencing was carried out on a NextSeq 550 machine (Illumina) using the NextSeq 500/550 High Output v2 Kit (Illumina), 75 cycles in single-end mode. After demultiplexing, Fastq files containing all the sequencing reads per sample were generated and used in downstream bioinformatics pipelines. Quality control was performed with FastQC v0.11.5. Adapters, 5’ and 3’ adjustments for possible end-repair bases and low-quality bases were removed using Trim Galore! v0.6.6 (FastQC) and Trim Galore! was downloaded from https://www.bioinformatics.babraham.ac.uk/. Bisulfite-treated reads were then aligned (using Bowtie v2.4.4) to the GRCm38/mm10 mouse reference genome followed by methylation calling using Bismark v0.22.3 22 (parameters for the mapping step: –non_bs_mm –bam –nucleotide_coverage; parameters for the methylation calls: –cytosine_report –comprehensive –merge_non_CpG).

All differential methylation analyses were performed between *Mtr*-cKO and WT groups under R 4.1 (RStudio v1.4.1106) with the MethylKit v1.16.1 package 23. The dataset was first filtered for low coverage (CpGs with coverage below 10x) and for extremely high coverage to exclude reads with PCR bias (CpGs with coverage more than the 99.9th percentile of coverage), then normalized following the median depth method, and finally merged by allowing one maximum missing position per group. After evaluation of the correlation structure between samples, differential CpGs were identified with the “calculateMethDiff” function, with false discovery rate (FDR) correction following the Benjamini–Hochberg procedure. The differentially methylated regions (DMRs) were identified within a window size of 2000 bp. DMRs were annotated using the mouse GRCm38/mm10 genome as a reference, with RefSeq curated (NCBI) and GENCODE VM22 (Ensembl) databases queried and prepared with the UCSC table browser (https://genome.ucsc.edu/).

### Bioinformatics analysis

We performed enrichment analyses on CpGs exhibiting increased or decreased methylation (methylation fold change > 25 and a cumulative *q*-value < 0.01) and on DMRs with a methylation fold change > 15, and a cumulative *q*-value < 0.01 using “Gene Ontology Biological Process” and/or “Reactome Pathway Database” to summarize statistically top ten significant terms based on their calculated FDR. Differentially methylated CpGs and DMRs are summarized on a volcano plot using GraphPad.

### Immunofluorescence cell imaging on flat-mounted retina

The eyes of 21-day-old mice, enucleated, are fixed with 4% PFA for 1 h at room temperature. After fixation, eyes were sectioned at the limbus, and then, the cornea and the lens were removed. The retinas were carefully separated from the choroid, and sclera. They were then washed in PBS and incubated in a 1 ml tube with a solution of primary antibodies (RBPMS #GTX118619, GENETEX; PNA #10,134,522 Fisher Scientific) diluted 1:100 in BSA-Triton overnight at 4 °C with gentle agitation. After incubation, the retinas were washed 3 times for 5 min with 1% PBS—Tween, at 4 °C and under slow agitation, and then incubated with 200μL of secondary antibody (Anti-Rabbit Abcam) diluted to 1/1000 and DAPI (Sigma) diluted to 1/3000 in BSA–Triton for 2 h at room temperature with stirring. The retinas were mounted flat between two coverslips with mounting fluid (Dako Fluorescent Mounting Medium) and observed under a confocal microscope (Nikon C2). For each retina, an image was recorded in 3 distinct zones: top, bottom, and right. The cell counting was performed using a blind manual technique by an external experimenter who was not aware of the phenotype to eliminate possible bias, using the Icy v2.4 software. For the retinal ganglion cells (RGCs), all the cells were counted and the number of RGCs was related to the number of total cells, while, for the cones, a rectangle with an area of 2 µm^2^ was used for counting. This rectangle was placed in the center-top, center, and center-bottom of each image, and then, the number of cones/mm^2^ was related to the number of nuclei.

### LC–MS/MS analyses

Briefly, each retina is placed in a 2 mL tube from the Lysing kit CK28 (Precellys, Cat. No.03961-1-002) along with ceramic beads. This combination facilitates efficient homogenization and lysis of the retinas. To initiate the process, 200 µL of PBS is added to the retinas. The tube is then inserted into a Precellys24 homogenizer (Bertin Instruments) operating at 5000 rpm for 15 s, followed by 5 s of rest. This cycle is repeated twice to ensure thorough processing. Once the retinas are homogenized, 16 µL of the resulting sample is used for protein quantification using BCA kit (Interchim). A standard curve is prepared, comprising a mixture of retinol, retinal, retinoic acid. Subsequently, 100 µL of acetonitrile (Dasit Group) is added to facilitate retinoid extraction. The tubes are vigorously vortexed to promote thorough mixing, followed by the addition of methyl-tert-butyl ether (MTBE) (Acros organics). Further vortexing is performed, and the tubes are then centrifuged for 10 min at 13,000 rpm followed by adding a solution of 75% methanol and 25% of formic acid. Experiments were carried out using Shimadzu LCMS 8045 ESI Triple quadrupoles (Kyoto, Japan) and analyzed using Insight software v3.1 (Shimadzu, Kyoto, Japan).

### RNA extraction and quantitative RT-PCR analysis

Five hundred nanograms of total RNA extracted for retina tissue using Nucleospin RNA plus kit (Macherey-Nagel) was subjected to 2-step RT-qPCR using PrimeScript™ RT Master Mix and SYBR® Premix Ex Taq™ (Takara, Saint-Germain-en-Laye, France) following the manufacturer’s instructions. Primers (Additional file 1: Table S2) were ordered from Eurogentec (Angers, France). Melting temperature was determined for each sample, and the expression of the genes of interest was normalized to those of *Tbp* and *Hprt* genes using BioRad CFX Maestro software.

### Protein analyses

#### Protein extraction and Western Blot/Wes Simple Protein analyses

Nitrogen-frozen retina samples were homogenized in RIPA lysis buffer complemented by phenylmethanesulfonyl fluoride 1% (Sigma), Sodium Orthovanadate 1% (Sigma), and Phosphatase Inhibitor Cocktail 0,5% (Roche). The protein concentration was determined using the BCA Protein Assay kit (Interchim) following the provider’s instructions. Considering the weak amount of total protein from the retina, analysis was performed using the Protein Simple Wes system (*ProteinSimple, USA*) or otherwise using classic western-blot experiments (in the case of failed compatibility between the used antibody and Wes). Western blot experiments were performed as described [38]. The membranes were incubated with Rhodopsin (#MAB5356, Merck Millipore) and RBPMS (#GTX118619, GENETEX) antibodies at 4 °C overnight. Peroxidase-labeled anti-rabbit (711-035-152, Interchim) or anti-mouse (715-035-152, Interchim) secondary antibodies were used at a 1:5000 dilution. The total amount of proteins per lane was normalized using α-tubulin (ab185067, Abcam), and densitometry analysis of the band intensity was achieved using ImageJ v1.53. The Wes automated capillary-based size sorting system (*ProteinSimple, USA*) was used for the analysis of protein expression. 0,4ug/uL of total protein was loaded to WES 25-well plates for separation (*Protein Simple, USA*) following provider instructions. Primary antibodies of MTR (#25,896-1-AP, Protein Tech), OPSIN (#AB5405, Millipore), and PAX6 (#ab5790, Abcam) were diluted at 1:75. The total amount of proteins per lane was normalized using α-tubulin (#2144, Cell Signaling). HRP-labeled anti-rabbit (*ProteinSimple, USA*) secondary antibodies were used at the provided concentration. The relative amount of each protein was analyzed through the areas under peaks from the chemiluminescence chromatograms by the Compass for SW software v6.0.0 (*ProteinSimple, USA*) with a virtual blot presentation.

#### Statistical analyses

Statistical analyses were performed under GraphPad Prism V10.0.2. Continuous variables and densitometry analyses were reported as means ± SEM. To analyze the data, the *Mtr*-cKO groups were compared using Student’s unpaired t test after confirming that the data followed Gaussian distributions and exhibited equal variances. Statistical significance was determined with a threshold of p value below 0.05, indicating significant differences between the compared groups.

### Supplementary Information

**Additional file 1**: **Table S1** Conversion rate of the methylated spike-in controls of RRBS Diagenode kit. **Table S2** Primers used for RT-qPCR analysis. **Fig. S1** RBPMS and Rhodopsin protein expression in the retina of *Mtr*-cKO vs Wild-type mice. **Fig. S2** Enhanced Genome Browser View panel revealing the Rara gene with ENCODE’s annotations.

## Data Availability

The data that support the findings of this study are available from the corresponding author, upon reasonable request.
