# Correction: A transgenic mice model of retinopathy of cblG‑type inherited disorder of one‑carbon metabolism highlights epigenome‑wide alterations related to cone photoreceptor cells development and retinal metabolism

**DOI:** 10.1186/s13148-024-01636-8

**Published:** 2024-02-08

**Authors:** Karim Matmat, Jean-Baptiste Conart, Paul-Henri Graindorge, Sandra El Kouche, Ziad Hassan, Youssef Siblini, Rémy Umoret, Ramia Safar, Okan Baspinar, Aurélie Robert, Jean-Marc Alberto, Abderrahim Oussalah, Sébastien Hergalant, David Coelho, Jean-Louis Guéant, Rosa-Maria Guéant-Rodriguez

**Affiliations:** 1 Inserm UMRS 1256 NGERE – Nutrition, Genetics, and Environmental Risk Exposure, University of Lorraine, 54500 Vandoeuvre-lès-Nancy, France; 2 National Center of Inborn Errors of Metabolism, University Regional Hospital Center of Nancy, 54000 Nancy, France; 3 Department of Ophthalmology, University Regional Hospital Center of Nancy, 54000 Nancy, France; 4 Faculté de Médecine, Bâtiment C 2Ème Étage, 9 Avenue de La Forêt de Haye, 54505 Vandœuvre-lès-Nancy, France

**Correction: Clinical Epigenetics (2023) 15:158** 10.1186/s13148-023-01567-w

Following publication of the original article [1], one of the co-author “Sébastien Hergalant“ was omitted in the authorship list. The correct authorship list is: Karim Matmat, Jean-Baptiste Conart, Paul-Henri Graindorge, Sandra El Kouche, Ziad Hassan, Youssef Siblini, Rémy Umoret, Ramia Safar, Okan Baspinar, Aurélie Robert, Jean-Marc Alberto, Abderrahim Oussalah, Sébastien Hergalant, David Coelho, Jean-Louis Guéant and Rosa-Maria Guéant-Rodriguez.

The author group has been updated and the affiliations have been updated.
