# Supplementary material for: A transgenic mice model of retinopathy of cblG-type inherited disorder of one-carbon metabolism highlights epigenome-wide alterations related to cone photoreceptor cells development and retinal metabolism
Source: Clin Epigenetics. 2023 Oct 5;15:158. doi: 10.1186/s13148-023-01567-w (PMC10557304; doi:10.1186/s13148-023-01567-w)
Supplement: Supplementary file 1 — Additional file 1: Table S1 Conversion rate of the methylated spike-in controls of RRBS Diagenode kit. Table S2 Primers used for RT-qPCR analysis. Fig. S1 RBPMS and Rhodopsin protein expression in the retina of Mtr-cKO vs Wild-type mice. Fig. S2 Enhanced Genome Browser View panel revealing the Rara gene with ENCODE’s annotations. [file 13148_2023_1567_MOESM1_ESM.docx]

**Supplementary table 1**

**Conversion rate of the methylated spike-in controls of RRBS Diagenode kit**

| Sample ID | Conv. Rate Meth. Spike-in (%) | Conv. Rate UnMeth. Spike-in (%) |
| --- | --- | --- |
| K39 | 1.53 | 99.35 |
| K95 | 1.37 | 99.41 |
| K96 | 1.40 | 99.23 |
| K97 | 1.58 | 99.30 |
| K99 | 1.28 | 99.20 |
| K100 | 1.30 | 99.31 |
| K116 | 1.23 | 99.22 |
| K118 | 1.51 | 99.40 |

**Supplementary table 2**

**Table of Primers used for RT-qPCR analysis**

|  |  |  |  |
| --- | --- | --- | --- |
| **Gene** | **Strand** | **Sequences** | **Tm (°C)** |
| *Mtr exon 4-6* | Forward | ACACTTGGCCTACCGGATG | 60 |
|  | Reverse | CCAGCCACAAACCTCTTGAC |  |
| *Lrat* | Forward | AAGCTCTTTAGCGTGAGCGT | 60 |
|  | Reverse | AAATGGGTCCGTGACACCTC |  |
| *Rdh5* | Forward | CTGGCTTCTTTCGAACCCCT | 60 |
|  | Reverse | TCGAAGATAAGTATCGAGGAAGGC |  |
| *Rpe65* | Forward | CCCTCCACTGAAAGCAGACAA | 60 |
|  | Reverse | AGTTGGCTCCCCAAAGACTC |  |
| *Rho* | Forward | CGCACACCCCTCAACTACAT | 60 |
|  | Reverse | AGGGCGATTTCACCTCCAAG |  |
| *Opn1mw* | Forward | TCCCACTCAGCATCATCGTG | 60 |
|  | Reverse | GAGGCAGTATGCGAAGACCA |  |
| *Rbpms* | Forward | GAAATCCCGCAAACGCTACG | 60 |
|  | Reverse | GCATATGGCTCCCTGGCAAT |  |
| *Tbp* | Forward | GCTCTGGAATTGTACCGCAG | 60 |
|  | Reverse | TGACTGCAGCAAATCGCTTG | 60 |
| *Pol2* | Forward | AGCAAGCGGTTCCAGAGAAG | 60 |
|  | Reverse | TCCCGAACACTGACATATCTCA | 60 |

**Supplementary figure 1**


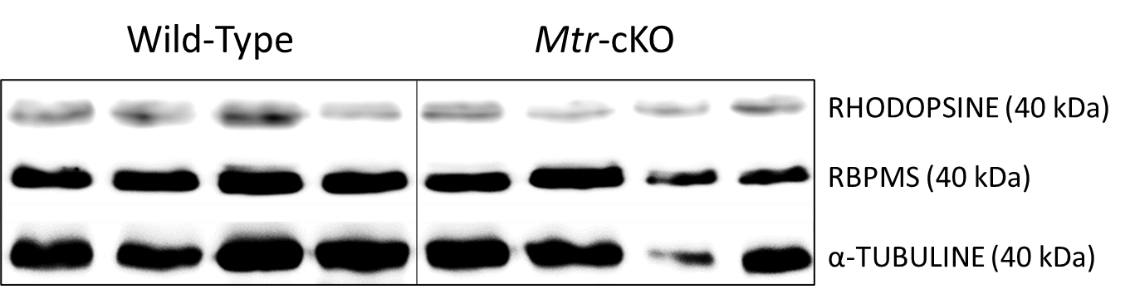


**Supplementary figure 2**


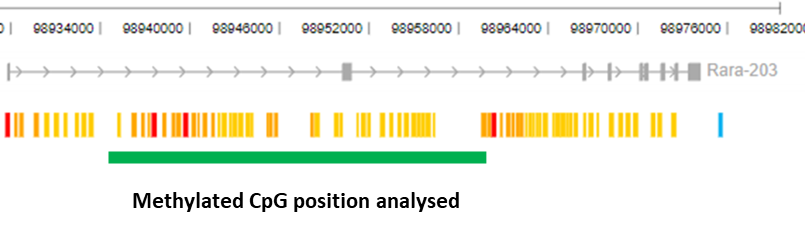


Enhanced Genome Browser View panel: Revealing the Rara gene with ENCODE's annotations. Orange: distal enhancers; Red: proximal enhancers. Green bar: region with differentially methylated CpG positions.
